# Enhanced Efficiency of Mixed-Halide Perovskite Solar Cells Through Optimization of the Layer Thicknesses, Defect Density, and Metal Contact Work Function

**DOI:** 10.3390/ma18071601

**Published:** 2025-04-01

**Authors:** Ezequiel Paz Totolhua, Jesús Carrillo López, José Álvaro David Hernández de la Luz, Karim Monfil Leyva, Javier Flores-Méndez, Ana Cecilia Piñón Reyes, Zaira Jocelyn Hernández Simón, José Alberto Luna López

**Affiliations:** 1 Centro de Investigaciones en Dispositivos Semiconductores (CIDS-ICUAP), Benemérita Universidad Autónoma de Puebla (BUAP), Col. San Manuel, Cd. Universitaria, Av. San Claudio y 14 Sur, Edificios IC5 y IC6, Puebla 72570, Mexico; e.paztotolhua@viep.com.mx (E.P.T.); jesus.carrillolopez@viep.com.mx (J.C.L.); jose.hernandez@correo.buap.mx (J.Á.D.H.d.l.L.); karim.monfil@correo.buap.mx (K.M.L.); zaira.hernandez@alumno.buap.mx (Z.J.H.S.); 2 Facultad de Ciencias de la Electrónica (FCE), Benemérita Universidad Autónoma de Puebla (BUAP), Col San Manuel, Cd. Universitaria, Av. San Claudio y 18 Sur, Edificio FCE1, Puebla 72570, Mexico; javier.floresme@correo.buap.mx; 3 Ingeniería Industrial, Tecnológico Nacional de México/I.T. Puebla, Av. Tecnológico No. 420, Col. Maravillas, Puebla 72220, Mexico; anacecilia.pinon@puebla.tecnm.mx

**Keywords:** mixed-halide PSCs, optimization, thickness, defect density, work function, SCAPS-1D software, power conversion efficiency

## Abstract

Mixed-halide perovskites enable the creation of high-performance and low-cost solar cells. Chloride incorporation enhances film morphology, carrier diffusion length, and stability, improving device performance. Nevertheless, optimizing film thickness, defect density, and metal contact work function remains insufficiently explored, despite its potential to enhance power conversion efficiency. In this study, a numerical simulation was performed using SCAPS-1D (version 3.3.10) to identify the optimal parameters for the FTO/TiO_2_/CH_3_NH_3_Pb_3−x_Cl_x_/Spiro-OMeTAD/Au configuration. The best performance parameters that have been published in the literature based on experimental results are as follows: *V_OC_* = 1.077 V, *J_SC_* = 21.45 mA/cm^2^, *FF* = 77.57%, and *PCE* = 17.97%. In contrast, the performance parameters obtained from numerical simulations for the same structure are *V_OC_* = 1.28 V, *J_SC_* = 21.63 mA/cm^2^, *FF* = 78%, and *PCE* = 21.53%. In our numerical analysis, we achieved efficiencies that were comparable to those reported in experimental studies, and after optimization, superior performance parameters were attained, including *V_OC_* = 1.179 V, *J_SC_* = 27.26 mA/cm^2^, *FF* = 81.03%, and *PCE* = 26.07%. These results indicate that optimized parameters can be integrated into the design and fabrication of mixed-halide perovskite solar cells to enhance performance.

## 1. Introduction

Fossil fuels such as coal, oil, and natural gas and are the most abundant and widely used energy sources for electricity production. The sustained utilization of these fuels has a significant impact on climate change and global warming [1]. Consequently, this has led to the use of alternative energy sources, especially renewable energies. In this context, solar energy is considered one of the most promising clean energy sources for the future due to its availability, purity, and inexhaustibility. Moreover, the use of solar energy is environmentally friendly [2]. Considering the global demand for sustainable energy solutions, perovskite solar cells (PSCs) have demonstrated noteworthy potential as photovoltaic technology due to their low costs, simple fabrication methods, and high efficiencies. Additionally, it should be noted that since their introduction, the power conversion efficiency (PCE) of PSCs has increased from 3.8% in 2009 to 25.6% within a comparatively small period [3,4].

The structure of perovskite materials is typically composed of three elements, with a general formula of ABX_3_, where A represents a monovalent organic, inorganic, or mixed cation such as methylammonium (MA^+^), formamidinium (FA^+^), or caesium (Cs^+^). B is a divalent cation, commonly lead (Pb^2^⁺) or tin (Sn^2^⁺), and X is a halide such as iodide (I⁻), bromide (Br⁻), or chloride (Cl⁻). Perovskite materials exhibit excellent properties, such as low exciton binding energy, long-range ambipolar charge transport, high absorption coefficients, long diffusion lengths, tuneable energy bandgaps, and low-temperature synthesis. These attributes make it an appropriate option for diverse optoelectronic devices, including photodetectors, LEDs, sensors, and solar cells [5].

MAPbX_3_ is the most widely used and studied perovskite material in PSCs for solar cell applications. Nevertheless, it has limited stability and environmental concerns restrict its applications [6]. Previous studies have shown that the incorporation of Cl⁻ in CH_3_NH_3_PbI_3_ perovskite improves the stability and crystallinity [7]. It has also been reported that the carrier lifetime, diffusion coefficient, and diffusion length are higher in mixed-halide perovskite (CH_3_NH_3_PbI_3−x_Cl_x_) compared to conventional perovskite (CH_3_NH_3_PbI_3_). The result is a better accumulation of charge carriers. This improves the performance of the fabricated device. Therefore, incorporating Cl⁻ ions into the perovskite is favourable for improving both stability and efficiency [8,9].

To enhance the PSCs efficiency, it is crucial to understand their structural design mechanisms. This can be achieved through the implementation of numerical simulations, using specialized simulation software programs [10]. Consequently, a comprehensive analysis of performance parameters, including power conversion efficiency (*PCE*), fill factor (*FF*), short-circuit current density (*J_SC_*), and open-circuit voltage (*V_OC_*), is imperative. This analysis should encompass changes in layer thickness, defect densities, series and parallel resistances, operating temperature, and the work function of the metal contact, to achieve high efficiency.

In this article, we simulated and optimized a PSCs (FTO/TiO_2_/CH_3_NH_3_PbI_3−x_Cl_x_/Spiro-OMeTAD/Au) using the SCAPS-1D software version 3.3.10. In the structure, Spiro-OMeTAD and TiO_2_ serve as the hole and electron transport layers (HTL and ETL, respectively). Although inorganic materials can be used as HTLs, which have advantages in their application such as stability, availability, and lower cost, this type of material shows significantly lower efficiency compared to HTLs made of organic materials. The most prominent organic HTLs are poly(3-hexylthiophene-2,5-diyl) (P3HT), poly[bis(4-phenyl) (2,4,6-trimethylphenyl) amine] (PTAA), PEDOT: PSS, and Spiro-OMeTAD. While all these materials have demonstrated promising results when they are used as HTLs, the best structures in terms of efficiency achieved (with efficiencies greater than 25%) are those that incorporate Spiro-OMeTAD [11].

In the context of organic ETLs, these materials typically exhibit an optimal alignment of the work function. However, their low conductivity requires the utilization of thin films with thicknesses less than 10 nm to achieve optimal performance, thereby limiting their large-scale application. In contrast, inorganic ETLs offer distinct advantages, including a low work function, stability, and cost-effectiveness in production. However, these materials are also characterized by an important number of surface defects, which cause interfacial problems with the active layer, resulting in a high recombination rate and series resistance [12]. Consequently, TiO_2_ emerges as a promising candidate for use as an ELT due to its inherent properties, including high mobility, excellent stability, and a wide band gap that facilitates charge separation. This material also exhibits a low trap density, further reducing the issues [13].

The simulation was performed by first optimizing the thicknesses of the absorbent layer, ETL, and HTL. Subsequently, we optimized the defect density in the absorber layer and the interfaces (ETL/Perovskite and Perovskite/HTL). Finally, the series and shunt resistances, work function of the metal contact, and the operating temperature were optimized. These results indicated that the key parameters for improving the performance of the mixed-halide PSC were the perovskite thickness, bulk defects in the perovskite, interfacial defects at ETL/Perovskite, and the work function of the metal contact. The proposed device FTO/TiO_2_/CH_3_NH_3_PbI_3−x_Cl_x_/Spiro-OMeTAD/Au achieved final performance parameters in the simulation of *V_OC_* = 1.179 V, *J_SC_* = 27.26 mA/cm^2^, *FF* = 81.03%, and *PCE* = 26.07%. These results were compared with experimental and simulation data, demonstrating that our optimized proposed structure exhibited superior performance. Finally, we obtained the J–V current density–voltage curve and the energy band diagram of the initial vs. optimized structure.

## 2. Materials and Methods

### 2.1. Solar Cell Structure

**Figure 1:**
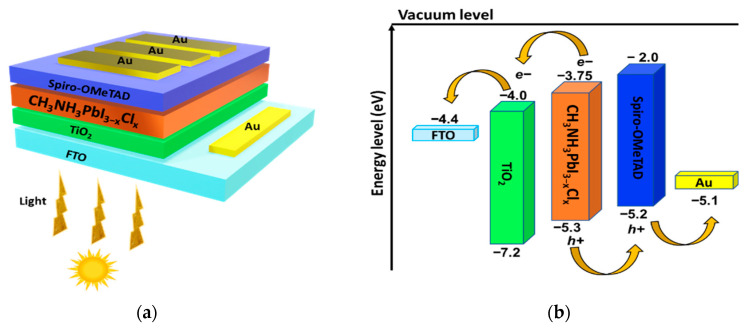
(**a**) Schematic diagram of the perovskite device structure and (**b**) energy bands alignment of the TCO, ETL, absorber, HTL, and back metal contact.

Figure 1a and Figure 1b illustrate the configuration and alignment of energy levels for the mixed-halide perovskite solar cell (FTO/TiO_2_/CH_3_NH_3_PbI_3−x_Cl_x_/Spiro-OMeTAD/Au), respectively. In the proposed design, fluorine-doped tin oxide (FTO) serves as the transparent conductive oxide (TCO) and functions as the front electrode; titanium dioxide (TiO_2_) acts as the electron transport layer (ETL); the mixed-halide perovskite material (CH_3_NH_3_PbI_3−x_Cl_x_) serves as the absorber layer; Spiro-OMeTAD functions as the hole transport layer (HTL); and gold (Au) is used as the back metal contact. In the structure, the generation of electron–hole pairs occurs in the perovskite absorber layer, which is responsible for generating the electric current. The photogenerated electrons are transported from the absorber layer to the ETL layer and collected by the FTO front contact. Similarly, the holes are transported from the absorber through the HTL layer and then collected by the gold electrode or back metal contact [14]. Additionally, Figure 1b illustrates the correct alignment of the energy levels of the materials in the PSC. According to the diagram, electrons tend to move to lower energy levels. The ETL has the lowest unoccupied (LUMO) and highest occupied (HOMO) molecular orbitals at lower energy levels compared to the perovskite. In contrast, the LUMO and HOMO of the HTL are located at higher energy levels than those of the perovskite, as shown in Figure 1b.

### 2.2. Basic Equations and Numerical Parameters

The simulation was performed using the one-dimensional solar cell capacitance simulator, SCAPS-1D, developed at the Department of Electronics and Information Systems (ELIS), Ghent University (Belgium). The software can solve the fundamental semiconductor equations in one dimension under steady-state conditions, including Poisson’s equation, continuity equations for electrons and holes, charge transport equations for electrons and holes, and the absorption coefficient [15,16,17]. The SCAPS-1D simulation is based on the following equations:

1. Poisson’s equation:

(1) $$\frac{d}{dx}(-\varepsilon (x)\frac{d\psi }{dx})=q\left[p\left(x\right)-n\left(x\right)+N_{D}^{+}\left(x\right)-N_{A}^{-}\left(x\right)+p_{t}\left(x\right)-n_{t}\left(x\right)\right]$$

where ε represents the electric field, ψ denotes the electrostatic potential, q is the elementary charge, and ε corresponds to the permittivity. Additionally, $N_{D}^{+}$ and $N_{A}^{-}$ denote the ionized donor and acceptor concentrations, respectively, while $n\left(x\right)$ and $p\left(x\right)$ represent the electron and holes densities. Furthermore, $n_{t}\left(x\right)$ and $p_{t}\left(x\right)$ correspond to the trapped electrons and holes as a function of x.

2. Continuity equations for electrons and holes:

(2) $$\;\;\;\frac{dJ_{n}}{dx}=G-R$$

(3) $$\;\;\;\;\frac{dJ_{p}}{dx}=G-R$$

Here, $J_{n}$ and $J_{p}$ represent the current densities of electrons and holes and G and R are the generation rate and recombination rate.

3. Charge transport equations for electrons and holes:

(4) $$J_{n}={q\mu }_{n}n\frac{d\psi }{dx}+{qD}_{n}\frac{dn}{dx}$$

(5) $$J_{p}={q\mu }_{p}p\frac{d\psi }{dx}+{qD}_{p}\frac{dp}{dx}$$

In these equations, $J_{n}$ and $J_{p}$ denote the current densities of electrons and holes, respectively, while $D_{n}$ and $D_{p}$ correspond to the diffusion coefficients of electrons and holes. Additionally, $\mu_{n}$ and $\mu_{p}$ represent the electron and hole mobilities.

4. Absorption coefficient:

(6) $$\alpha \left(\lambda \right)=\left(A+\frac{B}{h\nu }\right)\sqrt{h\nu -E_{g}}$$

In this equation, A and B are constants, $E_{g}$ is the bandgap of the absorber layer, h is Planck’s constant, and ν is the frequency of photons.

The optical and electrical parameters of the PSC materials were gathered from the scientific literature and are summarized in Table 1. Subsequently, Table 2 presents the defect density parameters and the electrical parameters of the back metal contact. The standard solar spectrum AM1.5 G was used for this simulation at 300 K.

## 3. Results and Discussion

### 3.1. The Perovskite Absorption Coefficient

**Figure 2:**
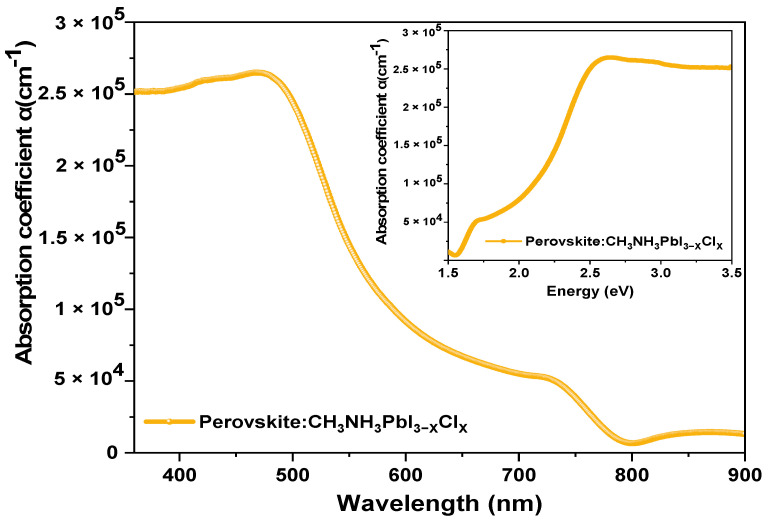
Experimental absorption coefficient for mixed-halide perovskite.

The absorption coefficient of the mixed-halide perovskite was experimentally determined and incorporated into the SCAPS-1D software. Figure 2 illustrates the absorption coefficient as a function of wavelength for the mixed-halide perovskite, while inset shows the absorption coefficient as a function of energy. It is evident that absorption is higher at shorter wavelengths (400 nm to 490 nm) and decreases at longer wavelengths (500 nm to 800 nm), with the peak observed at 730 nm.

### 3.2. Impact of the Perovskite Layer Thickness

The optimization of the perovskite thickness is essential because this is where light absorption and electron–hole pair generation occur. The variation of PCE with perovskite thickness is defined by increased absorption and the recombination rate. If the thickness is small, most of the incident light is transmitted. Therefore, increasing the thickness improves the absorption of the incident light, which results in a higher number of photogenerated carriers. However, if the thickness is too large, these photogenerated charge carriers must travel a longer distance, increasing the recombination rate and decreasing the lifetime of the carriers [35]. The decrease in *V_OC_* and *PCE* is mainly attributed to the increase in dark saturation current, which enhances carriers’ recombination when the thickness is too small or exceeds its optimum value. This behaviour can be explained by the dependence of the open-circuit voltage on the photogenerated current and the dark saturation current, as described by the following Equation (7) [36].

(7) $$V_{oc}=\frac{KT}{q}ln(\frac{J_{sc}}{J_{0}}+1)$$

where $\frac{KT}{q}$ is the thermal voltage, $J_{SC}$ is the photo-generated current density, and $J_{0}$ is the saturation current density.

**Figure 3:**
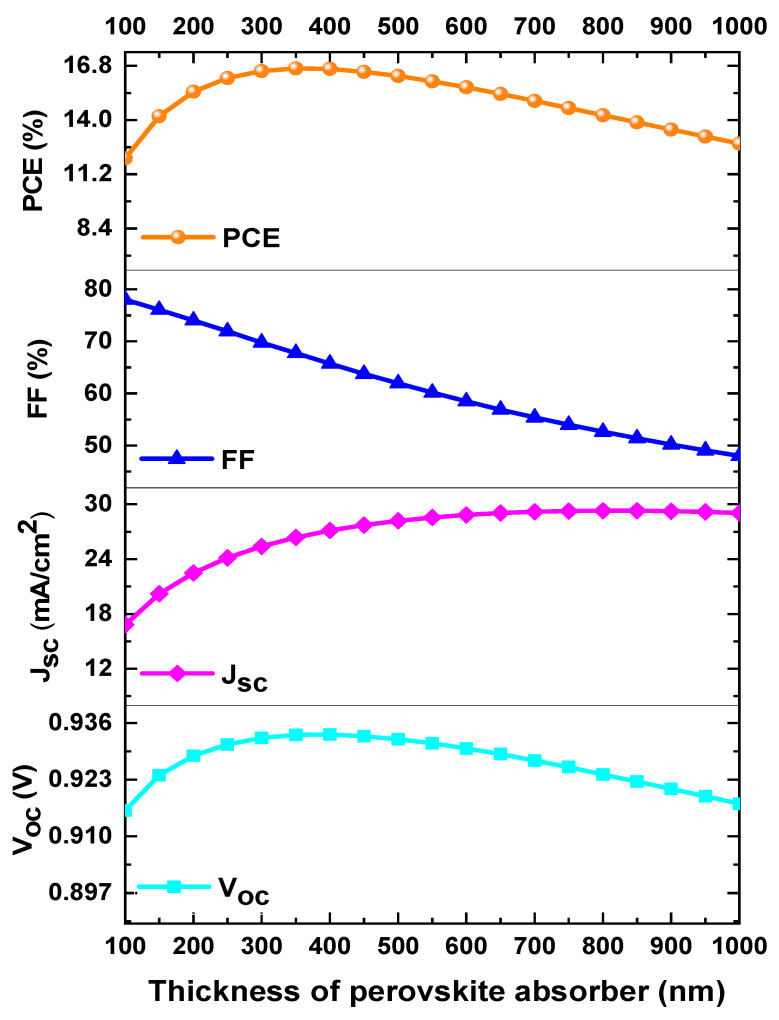
Variation of *PCE*, *FF*, *V_OC_*, and *J_SC_* as a function of the perovskite layer thickness of mixed halides.

Figure 3 shows the analysis of the effect of the absorber layer thickness on the performance parameters of the perovskite solar cell.

The perovskite thickness was varied from 100 to 1000 nm while keeping the other parameters constant during the simulation. Figure 3 shows the *PCE*, *FF*, *J_SC_*, and *V_OC_* as a function of the mixed-halide perovskite thickness. It was observed that the performance parameter *PCE* initially increased from 11.94% to 16.67% when the perovskite thickness changed from 100 to 350 nm. However, as the thickness was further increased (from 350 to 1000 nm), the *PCE* decreased from 16.67% to 12.89%. Meanwhile, the *FF* continuously decreased from 78.38% to 48.11%, while the *J_SC_* consistently increased from 16.54 mA/cm^2^ to 29.53 mA/cm^2^. Finally, the *V_OC_* increased from 0.9154 V to 0.9332 V as the thickness increased from 100 to 350 nm, after which it saturated and tended to slowly decrease when the thickness exceeded 350 nm, as shown in Figure 3. The optimized thickness of the mixed-halide perovskite in our analysis was 350 nm, and high-performance values were achieved.

### 3.3. Impact of the TiO_2_ Layer Thickness

**Figure 4:**
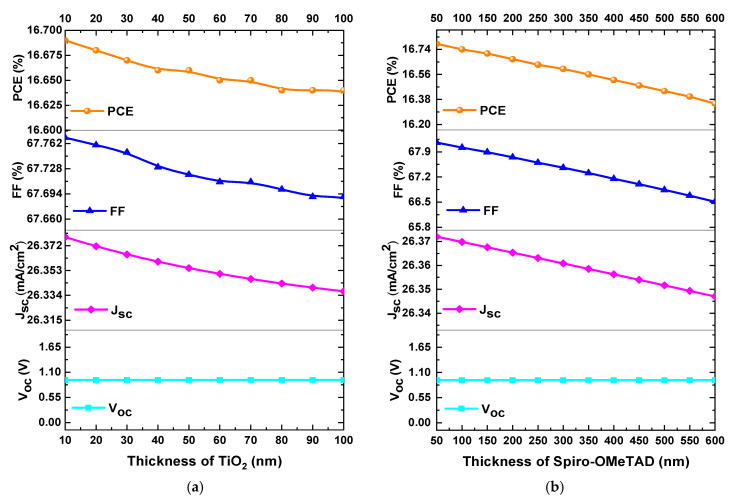
Variation of *PCE*, *FF*, *V_OC_*, and *J_SC_* as a function of (**a**) TiO_2_ thickness and (**b**) Spiro-OMeTAD thickness.

The ETL layer plays a crucial role in the performance of the perovskite solar cell, this layer not only transports electrons but also performs the following functions: (i) it acts as a scaffold for the perovskite layer, (ii) it blocks ultraviolet light from reaching the perovskite, (iii) it blocks hole transport, and (iv) it facilitates exciton dissociation at the TiO_2_/perovskite interface [37]. In this section, the effect of the TiO_2_ layer thickness on the performance parameters of the solar cell was analysed. The TiO_2_ thickness was varied from 10 to 100 nm while keeping the other parameters constant during the simulation. The results, depicted in Figure 4a, reveal the dependence of the *PCE*, *FF*, *J_SC_*, and *V_OC_* as a function of the TiO_2_ layer thickness.

As illustrated in Figure 4a, a reduction in *PCE* from 16.69% to 16.64%, *FF* from 67.77% to 67.69%, and *J_SC_* from 26.37 mA/cm^2^ to 26.33 mA/cm^2^ was observed, while the *V_OC_* value remained constant as the ETL thickness varied from 10 to 100 nm. It was observed that there were no significant changes in *PCE*, *FF*, *J_SC_*, and *V_OC_* as the TiO_2_ thickness increased. As the thickness of the TiO_2_ layer increases, the diffusion length and electron mobility decrease due to the higher defect density associated with the increased thickness, resulting in reduced *J_SC_* and *FF* values. Furthermore, as the TiO_2_ thickness increases, the irradiance on the perovskite absorber decreases. Consequently, lower irradiance on the perovskite absorber reduces the photogeneration of electron–hole pairs, leading to lower *J_SC_* and *PCE* values [38]. Therefore, an optimal ETL thickness of 45 nm was considered in our analysis, which provides optimal performance and meets the minimum thickness that can be experimentally deposited for this layer.

### 3.4. Impact of the Spiro-OMeTAD Layer Thickness

This section investigates the impact of HTL thickness on the performance parameters of the perovskite solar cell. High-efficiency PSCs typically incorporate Spiro-OMeTAD doped with lithium salts to enhance hole conductivity, with Spiro-OMeTAD deposited using the spin coating method. [39]. Generally, the HTL cannot absorb photons over a wide range like the perovskite layer, so the *V_OC_* value does not vary with changes in thickness and remains constant. It has been reported that the use of a thin layer of Spiro-OMeTAD leads to a significant enhancement in the *FF* value. This is attributed to the fact that holes require less travel distance, thereby reducing the impact of Spiro-OMeTAD conductivity on the device performance. However, it has been reported that devices with thin Spiro-OMeTAD layers exhibit reduced reproducibility, attributable to diminished uniformity of coverage on the rough perovskite layer. This allows for occasional direct contact between the perovskite and Au. As a result, these direct contacts increase the recombination rate of charge carriers, negatively impacting the efficiency of the solar cell [40]. In this section, the thickness of the HTL layer was varied from 50 to 600 nm, while keeping all other parameters constant throughout the simulation. Figure 4b illustrates the variations in *PCE*, *FF*, *J_SC_*, and *V_OC_* as a function of the Spiro-OMeTAD layer thickness. Figure 4b illustrates a decrease in the *PCE*, *FF*, and *J_SC_* values as the HTL thickness varies from 50 to 600 nm. For instance, the *PCE* value decreased from 16.78% to 16.35%, the FF decreased from 68.16% to 66.51%, and the *J_SC_* decreased from 26.37 mA/cm^2^ to 26.34 mA/cm^2^, while the *V_OC_* value remained constant. Given these results, there were no significant changes in *PCE*, *FF*, and *J_SC_* values as the thickness increased. Therefore, in our analysis, an optimal HTL layer thickness of 180 nm was considered, which provides the best performance parameters and represents the minimum thickness that can be experimentally deposited for this layer.

### 3.5. Impact of the Perovskite Layer Defect Density

One crucial factor in PSC research is the quality of the perovskite absorber layer. High density defects in the perovskite generate recombination centres, reducing the minority carrier lifetime and the carrier diffusion length, which ultimately decreases the power conversion efficiency [41,42]. On the other hand, maintaining a controlled deposition process for the perovskite layer prevents impurities and the formation of deep traps, thereby reducing the total defect density (*N_t_*). Consequently, it is advisable to consider low values of *N_t_* to achieve high device performance [43]. The defect density in the bulk of the perovskite is based on the Shockley–Read–Hall (SRH) recombination model, described by Equation (8) below [44].

(8) $$R_{SRH,Bulk}=\frac{np-{n_{i}}^{2}}{\tau_{p}\left(n+N_{c}e^{\left(\frac{E_{g}-E_{t}}{kT}\right)}\right)+\tau_{n}\left(p+N_{v}e^{\left(\frac{E_{t}}{kT}\right)}\right)}$$

where n represents the electron concentration, p represents the hole concentration, $n_{i}$ is the intrinsic carrier density, $E_{t}$ is the energy level of the defect density, and $\tau_{n,p}$ denotes the carrier lifetime.

As a result, the SRH model shows that an increase in carrier lifetime can reduce the recombination rate. The minority carrier lifetime ($\tau_{n,p}$) and the carrier diffusion length ($L_{D}$) can be obtained using Equations (9) and (10) [29].

(9) $$\tau_{n,p}=\frac{1}{N_{t}\delta v_{th}}$$

(10) $$L_{D}=\sqrt{\frac{\mu_{\left(n,\;p\right)}kT}{q}\tau_{n,p}}$$

where $\mu_{\left(n,\;p\right)}$ represents the mobility of electrons and holes, respectively, δ is the capture cross-sectional area for electrons and holes, $v_{th}$ is the thermal velocity of the carriers, and $N_{t}$ is the total defect density. As indicated these equations, an increase in $N_{t}$ within the perovskite bulk leads to the formation of recombination centres in the perovskite solar cell, which reduces the carrier diffusion length and decreases the carrier lifetime. Consequently, this directly impacts the reduction of the *V_OC_* and *PCE* values of the PSC [45].

**Figure 5:**
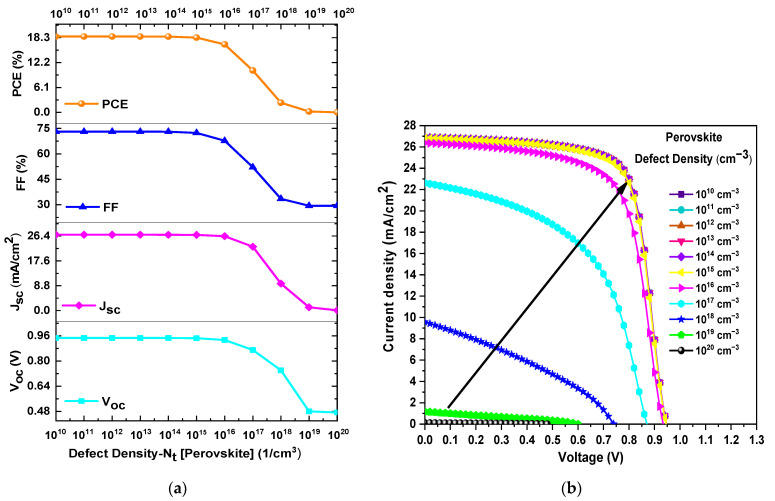
(**a**) Variation of *PCE*, *FF*, *V_OC_*, and *J_SC_* as a function of the *N_t_* of the perovskite layer and (**b**) J–V curves obtained from the *N_t_* variation of the perovskite layer.

In this section, the total defect density ($N_{t}$) of the mixed-halide perovskite was varied from 10^10^ a 10^20^ cm^−3^ while maintaining constant the other parameters during the simulation. Figure 5a presents the *PCE*, *FF*, *J_SC_*, and *V_OC_* as a function of $N_{t}$ in the perovskite layer. As observed in Figure 5a, there is a decrease in the values of *PCE*, *FF*, *J_SC_* and *V_OC_* as $N_{t}$ increases. For instance, *PCE* decreased from 18.57% to 0.02%, *FF* decreased from 73.08% to 29.35%, *J_SC_* decreased from 26.85 mA/cm^2^ to 0.101 mA/cm^2^, and *V_OC_* decreased from 0.946 V to 0.472 V when $N_{t}$ varied from 10^14^ cm^−3^ to 10^20^ cm^−3^. Furthermore, Figure 5b confirms that improved *J*–*V* curve output values can be obtained when $N_{t}$ is less than or equal to 10^14^ cm^−3^. Therefore, the optimal value of $N_{t}$ considered for the perovskite layer was 10^14^ cm^−3^.

### 3.6. Impact of the Defect Density of the Interfaces: TiO_2_/CH_3_NH_3_PbI_3−x_Cl_x_ and CH_3_NH_3_PbI_3−x_Cl_x_/Spiro-OMeTAD

The defect density at the interfaces also plays a crucial role in determining the performance of PSCs. The primary causes of defects at the interfaces (TiO_2_/CH_3_NH_3_PbI_3−x_Cl_x_ and CH_3_NH_3_PbI_3−x_Cl_x_/Spiro-OMeTAD) are associated with chemical impurities, uncoordinated atoms, dangling bonds, and surface dislocations that form on the perovskite surface during the deposition process [46]. Consequently, these defects act as recombination centres at the interfaces, degrading the performance parameters of PSCs [47]. The Shockley–Read–Hall (SRH) recombination for interface defects is determined by the surface recombination velocities of electrons and holes ($\delta_{n}$ and $\delta_{p}$). Therefore, the recombination rate at the interface can be expressed by Equation (11) [30].

(11) $$R_{SRH,interface}=\frac{n_{if}p_{if}-{n_{i}}^{2}}{\frac{p_{if}+N_{v}\;e^{\left(\frac{E_{t}}{kT}\right)}}{\delta_{n}}+\frac{n_{if}+N_{c}\;e^{\left(\frac{E_{g}-E_{t}}{kT}\right)}}{\delta_{p}}}$$

where $n_{if}$ and $p_{if}$ represent the concentrations of electrons and holes trapped at the interface, and $\delta_{n}$ and $\delta_{p}$ are the surface recombination velocities of electrons and holes, respectively.

The recombination at interface states is assessed based on the Paulwels–Vanhoutte theory, which extends the Shockley–Read–Hall (SRH) theory. The determination of recombination states at an interface can be achieved through a similar approach to that employed for the bulk of the perovskite. An ideal interface would minimize interfacial recombination losses (low values of $\delta_{n}$ and $\delta_{p}$), improving device performance [48].

**Figure 6:**
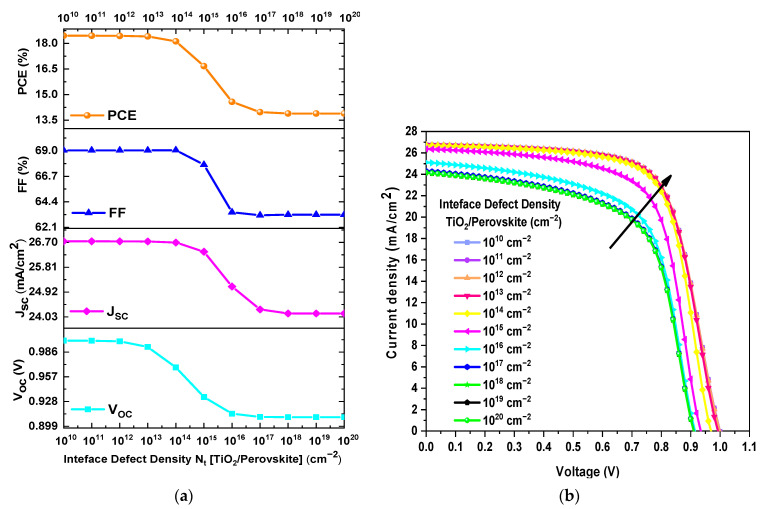
(**a**) Variation of *PCE*, *FF*, *V_OC_*, and *J_SC_* as a function of *N_t_* of the TiO2/CH3NH3PbI3−xClx interface and (**b**) J–V curves obtained from the *N_t_* variation of the interface.

In this section, the total defect density ($N_{t}$) at the interfaces was varied from 10^1^⁰ to 10^2^⁰ cm⁻^2^ while keeping the other parameters constant during the simulations. Figure 6a exhibits the *PCE*, *FF*, *J_SC_*, and *V_OC_* as a function of $N_{t}$ at the TiO₂/CH₃NH₃PbI_3−x_Clₓ interface. Figure 6a shows that the values of *PCE*, *FF*, *J_SC_*, and *V_OC_* decreased as the value of $N_{t}$ increased. For instance, *PCE* decreased from 18.41% to 13.89%, *FF* decreased from 69.04% to 63.24%, *J_SC_* decreased from 26.732 mA/cm^2^ to 24.152 mA/cm^2^, and *V_OC_* decreased from 0.9920 V to 0.9096 V when $N_{t}$ varied from 10^13^ cm⁻^2^ to 10^2^⁰ cm⁻^2^. Additionally, Figure 6b demonstrates that improved output values from the current density–voltage (J–V) curve are achieved when the $N_{t}$ value is equal to or less than 10^13^ cm⁻^2^. Consequently, the optimum value of $N_{t}$ for the TiO₂/CH₃NH₃PbI_3−x_Clₓ interface is when it has a value of 10^13^ cm^−2^.

**Figure 7:**
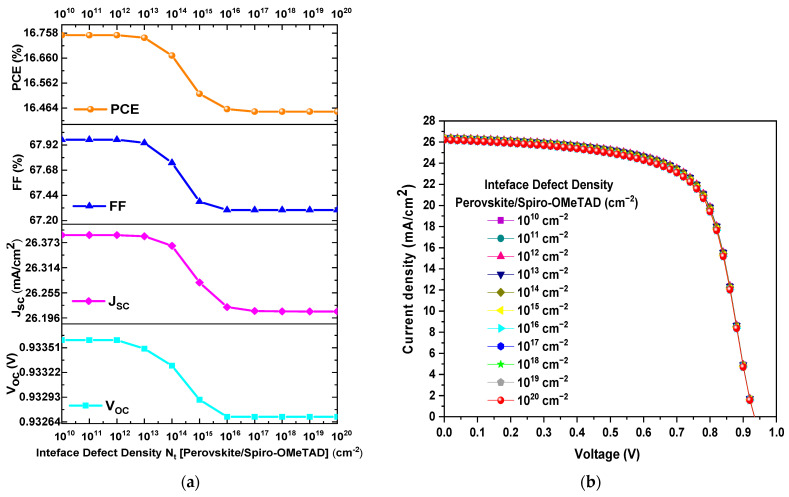
(**a**) Variation of *PCE*, *FF*, *V_OC_*, and *J_SC_* as a function of *N_t_* of the CH_3_NH_3_PbI_3−x_Cl_x_/Spiro-OMeTAD interface and (**b**) J–V curves obtained from the *N_t_* variation of the interface.

Figure 7a displays the *PCE*, *FF*, *J_SC_*, and *V_OC_* as a function of $N_{t}$ at the CH_3_NH_3_PbI_3−x_Cl_x_/Spiro-OMeTAD interface. It is evident from Figure 7a that there is a minimal decrease in the values of *PCE*, *FF*, *J_SC_*, and *V_OC_* as $N_{t}$ increases. For instance, *PCE* decreased from 16.74% to 16.45%, *FF* decreased from 67.94% to 67.30%, *J_SC_* decreased from 26.387 mA/cm^2^ to 26.211 mA/cm^2^, and *V_OC_* decreased from 0.933 V to 0.932 V when $N_{t}$ varied from 10^13^ cm^−2^ a 10^20^ cm^−2^. Furthermore, Figure 7b confirms that there is no significant change in the J–V curves when the value of $N_{t}$ increases or decreases. Therefore, the variation in the $N_{t}$ value at this interface does not introduce a significant change in the device performance parameters. However, it is noteworthy that the optimal $N_{t}$ value for the CH_3_NH_3_PbI_3−x_Cl_x_/Spiro-OMeTAD interface is when it has a value of 10^13^ cm^−2^.

### 3.7. Impact of the Series Resistance (R_series_) and Shunt Resistance (R_shunt_)

**Figure 8:**
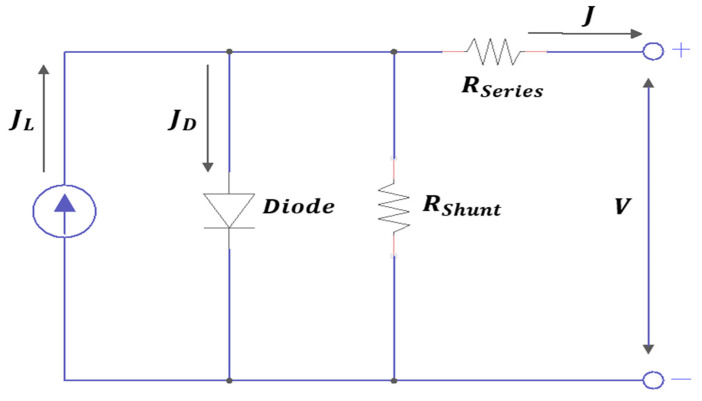
Equivalent electrical circuit diagram of a perovskite solar cell.

The series resistance (*R_series_*) and the shunt resistance *(R_shunt_*) have been demonstrated to have a significant impact on the efficiency of mixed-halide PSCs. These resistances determine the appearance and slope of the J–V characteristic plot. *R_series_* originates from the electrical resistance associated with the contacts (FTO and Au) and the electrical dissipation occurring in the ETL, HTL, and perovskite layers [28]. In addition, *R_shunt_* is associated with the manifestation of various recombination pathways and defects introduced during the film deposition process. Achieving a low *R_series_* value and a high *R_shunt_* value is essential for obtaining efficient PSCs [49]. Figure 8 shows the equivalent circuit of the perovskite solar cell under illumination. This circuit includes a current source associated with the lighting, a current source to perform charge separation (current generated by the diode), a series resistance (*R_series_*), and a shunt resistance *(R_shunt_*).

The J–V characteristic of the equivalent circuit can be expressed by Equation (12), which describes the influence of *R_series_* and *R_shunt_* on the performance of the solar device [43].

(12) $$J=J_{L}-J_{0}(e^{\left(q(V+J*R_{serie})/AkT\right)}-1)-\frac{V+J*R_{serie}}{R_{shunt}}$$

where J is the current through the external circuit, $J_{L}$ is the light-induced current density, $J_{0}$ is the saturation current density, V is the output voltage, A is the ideality factor, k is the Boltzmann constant, T is the temperature in Kelvin, and q is the electron charge. In an open-circuit condition (J≈0 mA/cm^2^), *V_OC_* and *R_shunt_* are expressed through equation (13).

(13) $$R_{shunt}=\frac{V_{oc}}{J_{L}-J_{0}\left[e^{\left[\frac{q(V_{oc})}{AkT}\right]}-1\right]\;}$$

Consequently, a high value of *R_series_* will mainly affect the *FF* and *J_SC_* values, while a low value of *R_shunt_* causes a loss of photovoltage and will affect the collected photocurrent [23].

**Figure 9:**
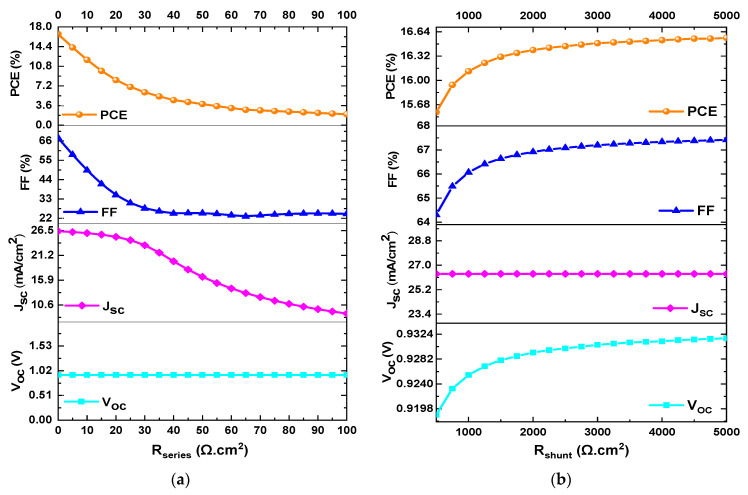
Variation of *PCE*, *FF*, *V_OC_*, and *J_SC_* as a function of (**a**) *R_series_* and (**b**) *R_shunt_*.

In this section, the effect of varying the values of *R_series_* and *R_shunt_* on the performance parameters of mixed-halide PSCs was observed. *R_series_* was varied from 0 $Ω\text{·}{\mathrm{cm}}^{2}$ to 100 $Ω\text{·}{\mathrm{cm}}^{2}$, while *R_shunt_* was varied from 500 $Ω\text{·}{\mathrm{cm}}^{2}$ to 5000 $Ω\text{·}{\mathrm{cm}}^{2}$. Figure 9a and Figure 9b illustrate the *PCE*, *FF*, *J_SC_*, and *V_OC_* as a function of *R_series_* and *R_shunt_*, respectively.

Regarding *R_series_*, *PCE* decreased from 16.67% to 2.01%, *FF* decreased from 67.75% to 24.53%, *J_SC_* decreased from 26.36 mA/cm^2^ to 8.78 mA/cm^2^, and *V_OC_* remained constant at a value of 0.9335 V when *R_series_* varied from 0 $Ω\text{·}{\mathrm{cm}}^{2}$ to 100 $\Omega \text{·}{\mathrm{cm}}^{2}$, as shown in Figure 9a. For *R_shunt_*, *PCE* increased from 15.58% to 16.56%, *FF* increased from 64.3% to 67.42%, *J_SC_* remained constant at a value of 26.365 mA/cm^2^, and *V_OC_* increased from 0.9188 V to 0.9317 V when *R_shunt_* varied from 500 $\Omega \text{·}{\mathrm{cm}}^{2}$ to 5000 $Ω\text{·}{\mathrm{cm}}^{2}$, as shown in Figure 9b. As a result, the optimal values considered to achieve high performance are when *R_series_* and *R_shunt_* have values of 1 $\Omega \text{·}{\mathrm{cm}}^{2}$ and 5000 $\Omega \text{·}{\mathrm{cm}}^{2}$, respectively.

### 3.8. Impact of the Operating Temperature and Metallic Contact Work Function

The operating temperature of the device plays a crucial role in the performance of mixed-halide PSCs. It is well-documented that perovskite exhibits degradation under conditions of elevated temperature and humidity. Furthermore, an increase in temperature induces deformations at the interfaces, leading to the formation of defects and, consequently, the creation of recombination centres. This phenomenon contributes to a reduction in the carrier diffusion length and lifetime. Additionally, an increase in temperature decreases the interconnectivity between layers [50]. These factors directly affect the *V_OC_* and PCE. As the temperature rises, the short-circuit current density (*J_SC_*) remains relatively constant; however, the *V_OC_* gradually decreases, thereby deteriorating the characteristics of the J–V curve. Consequently, the progressive reduction in *V_OC_* leads to a decline in the PCE value. Therefore, temperature variations directly influence the performance of PSCs through *V_OC_* [45,51]. The correlation between *V_OC_* and *T* is given by equation (14) below [45].

(14) $$\frac{d\left[V_{oc}\right]}{dT}=\frac{\left[V_{oc}-\frac{E_{g}}{q}\right]}{T}$$

In this equation, T denotes the operating temperature, $E_{g}$ is the energy bandgap, and q is the electron charge. According to the equation, the *V_OC_* differential is inversely proportional to the temperature. At higher temperatures, the mobility of electrons and holes, carrier concentrations, and energy bandgaps are affected, leading to diminished performance of the PSCs [52].

**Figure 10:**
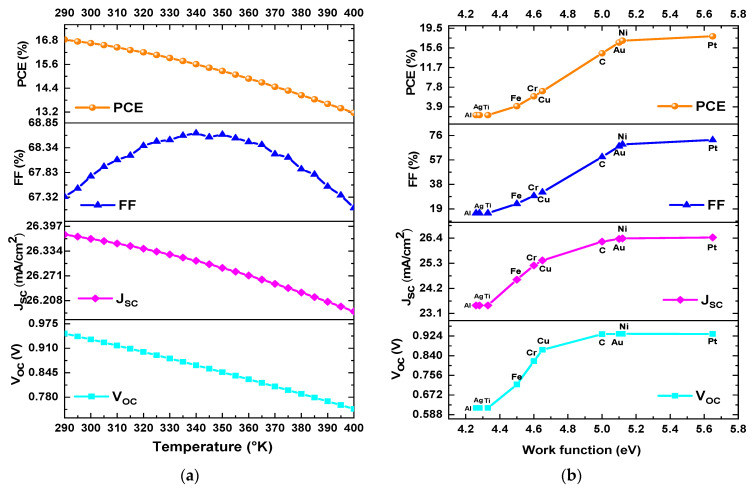
Variation of *PCE*, *FF*, *V_OC_*, and *J_SC_* as a function of (**a**) operating temperature and (**b**) back metal contact work function.

In this section, the effect of temperature on the performance parameters of mixed-halide PSCs is analysed. The temperature was systematically varied from 290 K to 400 K while maintaining constant all other parameters during the simulation. Figure 10a shows the *PCE*, *FF*, *J_SC_*, and *V_OC_* as a function of temperature. In Figure 10a a drop in *PCE* from 16.67% to 13.16% was observed. This decrease is associated with a corresponding reduction in *FF* and *V_OC_*. In addition, *FF* increased from 67.75% to 68.61% and then decreased to 67.1%, while *J_SC_* decreased from 26.36 mA/cm^2^ to 26.18 mA/cm^2^, remaining nearly constant. Finally, *V_OC_* decreased from 0.933 V to 0.748 V as the temperature varied from 300 K to 400 K. The best efficiency is at a lower temperature of 300 K.

The working function of the metal back contact can be crucial in improving or reducing the performance of a PSC. In mixed-halide PSCs, different materials could be used as back metal contacts. Some of the materials used as metal contacts in the simulation were as follows: silver (Ag = 4.26 eV), aluminium (Al = 4.28 eV), titanium (Ti = 4.33 eV), iron (Fe = 4.5 eV), chromium (Cr = 4.6 eV), copper (Cu = 4.65 eV), carbon (C = 5.0 eV), gold (Au = 5.1 eV), nickel (Ni = 5.12 eV), and platinum (Pt = 5.65 eV) [34,53].

Figure 10b shows the *PCE*, *FF*, *J_SC_*, and *V_OC_* as a function of the work function of various materials used as back contacts. In the Figure 10b, as the work function of the contact increased, the values of *PCE*, *FF*, *J_SC_*, and *V_OC_* also increased. According to the literature, a higher work function of the metal contact increases the built-in voltage (*V_bi_)* and consequently improves the efficiency. However, a reduced work function value corresponds to diminished efficiency, as the electric field in proximity to the HTL/metal interface becomes negative. This is due to the propensity of holes to migrate toward the electrode [54]. In this simulation analysis, the highest PCE obtained for the mixed halide PSC was achieved with a work function of 5.1 eV, 5.12 eV, and 5.65 eV, corresponding to Au, Ni, and Pt, respectively. The objective of this study was to use gold as the back metal contact because it allows for higher efficiencies and is more stable material. Conversely, nickel emerged as a cost-effective alternative, exhibiting a lower cost but compromised stability in PSC fabrication. Platinum is another viable option; however, its cost is significantly higher [53].

### 3.9. J–V Characteristic Curve, EQE, and Energy Band Diagram

**Figure 11:**
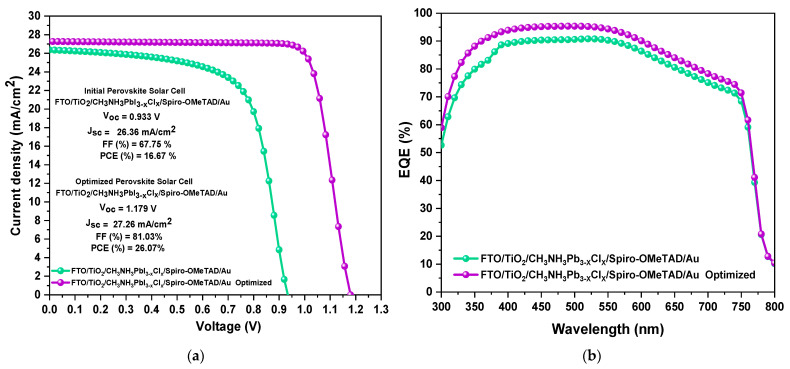
(**a**) J–V characteristic curve and (**b**) EQE curve of the non-optimized mixed-halide PSC vs. the op-timized mixed-halide PSC.

This section analyses the J–V characteristic curve, external quantum efficiency (EQE), and energy band diagram comparing the non-optimized and optimized mixed-halide perovskite solar cells (PSCs) simulated in SCAPS-1D. The initial mixed-halide PSC with the FTO/TiO_2_/CH_3_NH_3_PbI_3−x_Cl_x_/Spiro-OMeTAD/Au structure exhibited output parameters of *V_OC_* = 0.933 V, *J_SC_* = 26.36 mA/cm^2^, *FF* = 67.75%, and *PCE* = 16.67%. In contrast, after optimization, the same structure achieved enhanced performance with *V_OC_* = 1.179 V, *J_SC_* = 27.26 mA/cm^2^, *FF* = 81.03%, and *PCE* = 26.06%, as illustrated in Figure 11a. The simulation of the final mixed-halide PSC was conducted by incorporating the optimal parameters derived from the previous simulations. The optimized device exhibited an efficiency 9.4% higher than the initial device, demonstrating that the perovskite thickness, perovskite defects, TiO_2_/CH_3_NH_3_PbI_3−x_Cl_x_ interface defects, and work function were the primary factors contributing to the performance improvement of the Cl-based PSC.

Then, we obtained the external quantum efficiency (EQE) plot for both devices, as illustrated in Figure 11b. The EQE is the ratio between the total extracted charge carries in the PSC and the total incident photons. For this analysis, the EQE of both devices was simulated over a wavelength range of 300 to 800 nm. It can be observed (Figure 11b) that the non-optimized PSC exhibits an average EQE of 89% within the 380–600 nm wavelength range. On the other hand, the optimized PSC shows an average EQE of 94% within the 380–600 nm range, after which the EQE tends to decrease at longer wavelengths. Therefore, the increase in EQE is due to the incorporation of optimal parameters in the simulation, which enhances charge carrier extraction in the device.

**Figure 12:**
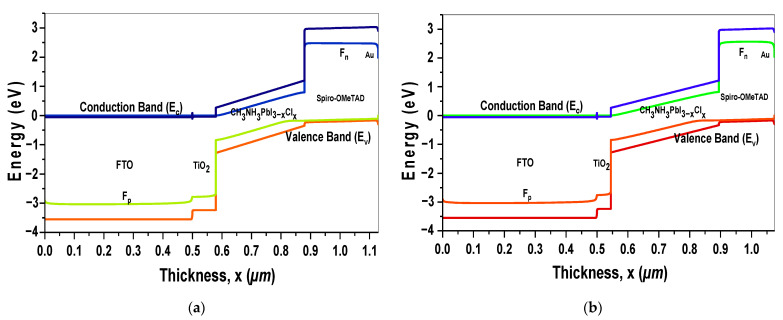
(**a**) Energy band diagram of the non-optimized mixed-halide PSC and (**b**) the energy band dia-gram of the optimized mixed-halide PSC.

Subsequently, Figure 12a,b show the simulated energy band diagrams of the initial mixed-halide PSC and the optimized mixed-halide PSC. In the energy band diagram of the optimized PSC, a splitting of the valence band and conduction band is observed, and changes in the thicknesses of the layers (Perovskite, ETL, and HTL) are observed due to optimization. Furthermore, the diagram illustrates that the energy band gaps of both the TiO_2_ and Spiro-OMeTAD layers are higher than that of the perovskite layer (CH_3_NH_3_PbI_3−x_Cl_x_), leading to effective energy level alignment. This alignment enhances the transport of electrons and holes generated within the perovskite layer while blocking the movement of opposite charges. Consequently, this also enables maximum photon transmission into the perovskite layer, generating more current, improving the *FF*, and enhancing the *PCE*.

### 3.10. Comparative Study Between the Experiment and Simulation

Table 3 presents a comparative analysis of the analogous mixed-halide PSC structures reported. This table includes both experimental reports and simulation studies. These structures are selected because they are like the structure proposed in this work. Compared to experimental reports, our optimized simulation proposal exhibits significantly superior performance.

## 4. Conclusions

In this analysis, we utilized the SCAPS-1D software to simulate and numerically optimize a mixed-halide perovskite solar cell with the structure FTO/TiO_2_/CH_3_NH_3_PbI_3−x_Cl_x_/Spiro-OMeTAD/Au. In addition, the experimentally obtained absorption coefficient of the perovskite layer was introduced into the simulator. Initially, the optimal thickness for the perovskite layer was determined to be 350 nm, while the optimal thicknesses for the ETL and HTL layers were 45 nm and 180 nm, respectively. Subsequently, the optimal defect densities for the perovskite layer and interfaces were 10^14^ cm^−3^ and 10^13^ cm^−2^, respectively. Finally, the optimal values for series resistance, shunt resistance, operating temperature, and work function of the metallic contact (gold) were 1 Ω·cm^2^, 5000 Ω·cm^2^, 300 K, and 5.1 eV, respectively. The simulation of the initial device produced performance parameters of *PCE* = 16.67%, *FF* = 67.75%, *J_SC_* = 26.36 mA/cm^2^, and *V_OC_* = 0.933 V. In contrast, the optimized simulation yielded improved performance parameters of *PCE* = 26.07%, *FF* = 81.03%, *J_SC_* = 27.26 mA/cm^2^, and *V_OC_* = 1.179 V. The analysis indicates that the primary factors contributing to the enhancement of performance are perovskite thickness, perovskite defects, ETL/perovskite interface defects, and back metal contact work function. A comparison of J–V characteristic curves between the initial and optimized devices revealed a voltage increase of 0.246 V, a rise in current density of 0.9 mA/cm^2^, and a 9.4% enhancement in efficiency. Regarding EQE, an increase of 5% was observed in the wavelength range of 380 to 600 for the optimized device compared to the non-optimized device. Ultimately, this study could be valuable for the fabrication of mixed-halide perovskite solar cells with a conventional architecture.

## Figures and Tables

**Table 1:** Parameters of the different materials used for simulation.

Parameters	FTO (TCO) [18,19,20]	TiO_2_ (HTL) [21,22,23]	Perovskite (CH_3_NH_3_PbI_3−x_Cl_x_) [24,25,26]	Spiro-OMeTAD (ETL) [27,28,29]
Thickness (nm)	500	80	300	250
$\mathrm{Band}\;\mathrm{gap},\;E_{g}\;(\mathrm{eV})$	3.5	3.2	1.55	3.2
Electron Affinity, χ (eV)	4.0	4.2	3.9	2.1
Relative permittivity, ε	9.0	9.0	30	3
$\mathrm{Effective}\;\mathrm{CB}\;\mathrm{density}\;\mathrm{of}\;\mathrm{states},\;N_{c}\;(1/{\mathrm{cm}}^{3})$	$2.2\;\text{×}{\;10}^{18}$	$2.0\;\text{×}{\;10}^{18}$	$2.2\;\text{×}{\;10}^{18}$	$2.2\;\text{×}{\;10}^{18}$
$\mathrm{Effective}\;\mathrm{VB}\;\mathrm{density}\;\mathrm{of}\;\mathrm{states},\;N_{v}\;(1/{\mathrm{cm}}^{3})$	$2.2\;\text{×}{\;10}^{19}$	$1.8\;\text{×}{\;10}^{19}$	$1.8\;\text{×}{\;10}^{19}$	$1.8\;\text{×}{\;10}^{19}$
$\mathrm{Electron}\;\mathrm{thermal}\;\mathrm{velocity},\;V_{n}\;(\mathrm{cm}/s)\;$	$1.0\;\text{×}{\;10}^{7}$	$1.0\;\text{×}{\;10}^{7}$	$1.0\;\text{×}{\;10}^{7}$	$1.0\;\text{×}{\;10}^{7}$
$\mathrm{Hole}\;\mathrm{thermal}\;\mathrm{velocity},\;V_{p}\;(\mathrm{cm}/s)\;$	$1.0\;\text{×}{\;10}^{7}$	$1.0\;\text{×}{\;10}^{7}$	$1.0\;\text{×}{\;10}^{7}$	$1.0\;\text{×}{\;10}^{7}$
$\mathrm{Electron}\;\mathrm{mobility},\;\mu_{n}\;({\mathrm{cm}}^{2}/\mathrm{Vs})$	20	20	2	0.0002
$\mathrm{Hole}\;\mathrm{mobility},\;\mu_{p}\;({\mathrm{cm}}^{2}/\mathrm{Vs})$	10	10	2	0.0002
$\mathrm{Donor}\;\mathrm{concentration},\;N_{D}\;(1/{\mathrm{cm}}^{3})$	$2.0\;\text{×}{\;10}^{19}$	$1.0\;\text{×}{\;10}^{19}$	$1.0\;\text{×}{\;10}^{13}$	0
$\mathrm{Acceptor}\;\mathrm{concentration},\;N_{A}\;(1/{\mathrm{cm}}^{3})$	0	0	$1.0\;\text{×}{\;10}^{13}$	$2.0\;\text{×}{\;10}^{18}$
$\mathrm{Defect}\;\mathrm{density},\;N_{t}\;(1/{\mathrm{cm}}^{3})$	$1.0\;\text{×}{\;10}^{15}$	$1.0\;\text{×}{\;10}^{15}$	$1.0\;\text{×}{\;10}^{17}$	$1.0\;\text{×}{\;10}^{15}$

**Table 2:** Parameters for defects and back metal contact used for simulation.

Parameters	Perovskite (CH_3_NH_3_PbI_3−x_Cl_x_) [30,31]	HTL/Perovskite [30,31]	Perovskite/ETL [30,31]
Defect type Electron capture cross section (cm^2^) Hole capture cross section (cm^2^) Energetic distribution Reference for defect energy level E_t_ Energy level with respect to Reference (eV) Total density (integrated over all energies) (1/cm^3^)	Neutral 1.0 × 10^−15^ 1.0 × 10^−15^ Gaussian Below E_c_ 0.65 1.0 × 10^17^	Neutral 1.0 × 10^−15^ 1.0 × 10^−15^ Single Above E_V_ 0.6 1.0 × 10^15^	Neutral 1.0 × 10^−15^ 1.0 × 10^−15^ Single Above E_V_ 0.6 1.0 × 10^15^
**Parameters**	**Gold (Au)** **[32,33,34]**		
Work function (eV) Surface recombination velocity of electrons (cm/s) Surface recombination velocity of holes (cm/s)	5.1 10^7^ 10^5^		

**Table 3:** Experimental and simulated comparison of mixed-halide PSC structures.

Device Structure	$V_{\mathrm{OC}}$ (V)	$J_{\mathrm{SC}}$ (mA/cm^2^)	FF (%)	PCE (%)	Ref.
FTO/TiO_2_+Al_2_O_3_/CH_3_NH_3_PbI_3−x_Cl_x_/Spiro-OMeTAD/Au (Experimental)	1.02	21.5	71	15.9	[55]
ITO/TiCl-TiO_2_/CH_3_NH_3_PbI_3−x_Cl_x_/Spiro-OMeTAD/Au (Experimental)	1.09	19.7	75.9	16.4	[56]
ITO/У-TiO_2_/CH_3_NH_3_PbI_3−x_Cl_x_/Spiro-OMeTAD/Au (Experimental)	1.077	21.45	77.57	17.91	[57]
FTO/TiO_2_/CH_3_NH_3_PbI_3−x_Cl_x_/Spiro-OMeTAD/Au (Numerical simulation)	1.28	21.63	78	21.53	[26]
FTO/TiO_2_/CH_3_NH_3_Pb_3−x_Cl_x_/Spiro-OMeTAD/Ag (Experimental)	1.25	26.11	69.89	22.72	[24]
FTO/TiO_2_/CH_3_NH_3_PbI_3−x_Cl_x_/Spiro-OMeTAD/Au (Initial numerical simulation)	0.933	26.36	67.75	16.67	This work
FTO/TiO_2_/CH_3_NH_3_PbI_3−x_Cl_x_/Spiro-OMeTAD/Au (Optimized numerical simulation)	1.179	27.26	81.03	26.07	This work

## Data Availability

The original contributions presented in this study are included in the article. Further inquiries can be directed to the corresponding author.
